# Redescription of *Cichlidogyrus philander* (Monogenea, Ancyrocephalidae) using scanning electron microscopy (SEM) and molecular analysis

**DOI:** 10.1051/parasite/2017046

**Published:** 2017-12-01

**Authors:** Patience C Igeh, Quinton M Dos Santos, Annemariè Avenant-Oldewage

**Affiliations:** a Department of Zoology, University of Johannesburg, PO Box 524, Auckland Park 2006, Johannesburg South Africa; b Department of Microbiology, Plateau State University, PO Box 2012, Jos Nigeria

**Keywords:** *Pseudocrenilabrus philander*, sclerites, molecular analysis, South Africa, Padda Dam

## Abstract

The sclerotized structures of monogeneans have traditionally been studied by light microscopy and different staining techniques. Recently, enzymatic digestion followed by scanning electron microscopy (SEM) has enabled the examination of structural details not visible with light microscopy. In order to obtain better, and more accurate, morphological information on sclerotized structures not affected by mounting medium or cover slip pressure, the sclerites of *Cichlidogyrus philander* Douëllou, 1993 (Monogenea, Ancyrocephalidae), collected from *Pseudocrenilabrus philander* (Weber, 1897), were redescribed using SEM. Parasites were collected from Padda Dam, Gauteng, South Africa and soft tissue was digested to release the sclerotized structures. The digested tissue also provided sufficient genetic material for molecular characterization of this species. *Cichlidogyrus philander* is characterised by a penis with a sharp, curved, lateral termination, an accessory piece with a hook-like extremity that may appear forked terminally, and lack of a visible vagina. The transverse bars have concave and convex surfaces with ribs on the concave surface. The dorsal bar bears fenestrations at the base of the auricles and the ventral and dorsal gripi are dissimilar. Furthermore, the large first pair of uncinuli shows lateral wings on the left side of the base. On top of this wing, a ball-like structure with a small fenestration is visible. Genetic characters derived from the 28S rDNA, the COI mitochondrial DNA and ITS1 rDNA regions distinguish *C. philander* from all other *Cichlidogyrus* sequenced species.

## Abstract (fr)

Les structures sclérifiées des monogènes ont traditionnellement été étudiées par microscopie optique et différentes techniques de coloration. Récemment, la digestion enzymatique suivie par la microscopie électronique à balayage (MEB) a permis d'examiner des détails structuraux non visibles en microscopie optique. Les sclérites de *Cichlidogyrus philander* Douëllou, 1993 (Monogenea, Ancyrocephalidae), collecté chez *Pseudocrenilabrus philander*, sont redécrites par MEB afin d'obtenir une information morphologique meilleure et plus précise des structures sclérifiées, non affectée par le milieu de montage. Les parasites ont été recueillis à Padda Dam, Gauteng, Afrique du Sud et les tissus mous ont été digérés pour libérer les structures sclérifiées. Le tissu digéré a fourni également suffisamment de matériel génétique pour la caractérisation moléculaire de l'espèce. *Cichlidogyrus philander* se caractérise par un pénis avec une terminaison latérale courbée et pointue, une pièce accessoire avec une extrémité en forme de crochet qui peut apparaître fourchue terminalement, et l'absence de vagin visible. Les barres transversales ont des surfaces concaves et convexes avec des nervures sur les surfaces concaves. La barre dorsale porte des fenestrations à la base des oreillettes et les gripi ventraux et dorsaux sont dissemblables. En outre, la première grande paire d'uncinuli montre des ailes latérales sur le côté gauche de la base. Au sommet de cette aile, une structure en forme de boule avec une petite fenestration est visible. Les caractères génétiques dérivés de l'ADNr 28S, de l'ADN mitochondrial COI et de l'ADNr ITS1 distinguent *C. philander* de toutes les autres espèces séquencées de *Cichlidogyrus*.

## Introduction

*Cichlidogyrus philander* Douëllou, 1993 is a monogenean ectoparasite occurring on the gills of *Pseudocrenilabrus* *philander* (Weber, 1897). It was first described by Douëllou from Lake Kariba, Zimbabwe [3]. The species was later found in the Padda Dam, Gauteng, South Africa [15,16] and apart from these two sites, *C. philander* has not been documented in any other locality. *Cichlidogyrus* Paperna, 1960 includes more than 100 species and has a wide distribution [13]. Like other monogeneans, the members of this genus use a specialized, posteriorly situated organ, the haptor, to attach themselves to the host [24,25]. In *Cichlidogyrus,* the haptor is composed of hooks and transverse bars [25,27] and these structures form a functional unit and have adapted to specific sites (microenvironment) within their host [37].

In monogenean morphological taxonomy, copulatory organs and haptoral parts have been widely used for identification and remain a key diagnostic feature [14,20]. In most monogeneans, the morphology of the haptoral sclerites is used for genus determination and the morphology of the male apparatus (comprising a penis and accessory piece) for species identification [28]. These parts have traditionally been studied using light microscopy and various staining techniques, enabling morphological descriptions and morphometry. However, classic techniques based on light microscopy do not allow detailed examination of this sclerotized structure [2]. The fixation and preparation, as well as the amount of pressure applied to the coverslip, may interfere with the interpretation and measurement of these structures [2,6,22]. An example is found in Fankoua *et al*. [6], where they mentioned that the type of fixative/preservative and mounting medium used has an effect on the size and shape of monogenean sclerites. They observed that the use of Hoyer's medium clears tissue, softens the sclerites and makes them lie flat under the cover slip pressure but on the contrary, the sclerites become enlarged and deformed, which does not accurately reflect the shape and size of these structures. Mo and Appleby found that sclerotized parts could be exposed by enzymatic digestion and subsequently examined by scanning electron microscopy (SEM) [22]. Subsequent authors modified the method in various ways [2,7,10,11,31,32]. Shinn *et al.* mentioned that, in addition to releasing the sclerites from the tissue, digestion makes it possible for the sclerites to lie flat, allowing more accurate visualizations and measurements [31]. The original description of *C. philander* was limited to light microscopy [3]. In this study, the sclerotized parts of *C. philander* are redescribed using SEM for the purpose of obtaining better and more accurate data on the morphology of the sclerotized structures, not affected by mounting medium or cover slip pressure, to provide additional information on *C. philander*. Furthermore, a molecular analysis is performed to examine the distinctiveness of this taxon.

## Materials and methods

### Collection of fish and parasites

Following approval from the Ethics Committee of the University of Johannesburg's Faculty of Science, and obtaining a permit from Nature Conservation in Gauteng, South Africa (permit numbers: CPE2-000116 and CPE3-000134), 20 *P. philander* were captured by electro-narcosis and hand nets from Padda Dam (20°10ʹS; 17°59ʹE), which is located on University of Johannesburg grounds, Gauteng. Captured fish were transported to the laboratory where they were kept in a holding tank with aerated dam water. Each fish was then weighed, measured and euthanised by a single cut through the spinal cord. The gills were removed using dissection scissors and tweezers and examined with a Zeiss stemi 350 compact-stereomicroscope. The parasites were gently removed with a preparation needle.

### Morphological study

Collected parasites were fixed in 70% ethanol and mounted individually in a drop of glycerine-ammonium-picrate (GAP) on a slide [18]. The preparation was then covered with a coverslip and sealed with nail varnish. Some specimens were stained with Horen's trichrome as described in the Manual of Veterinary Parasitological Techniques [19], cleared, mounted in a drop of lactophenol and sealed with nail varnish.

**Figure 1:**
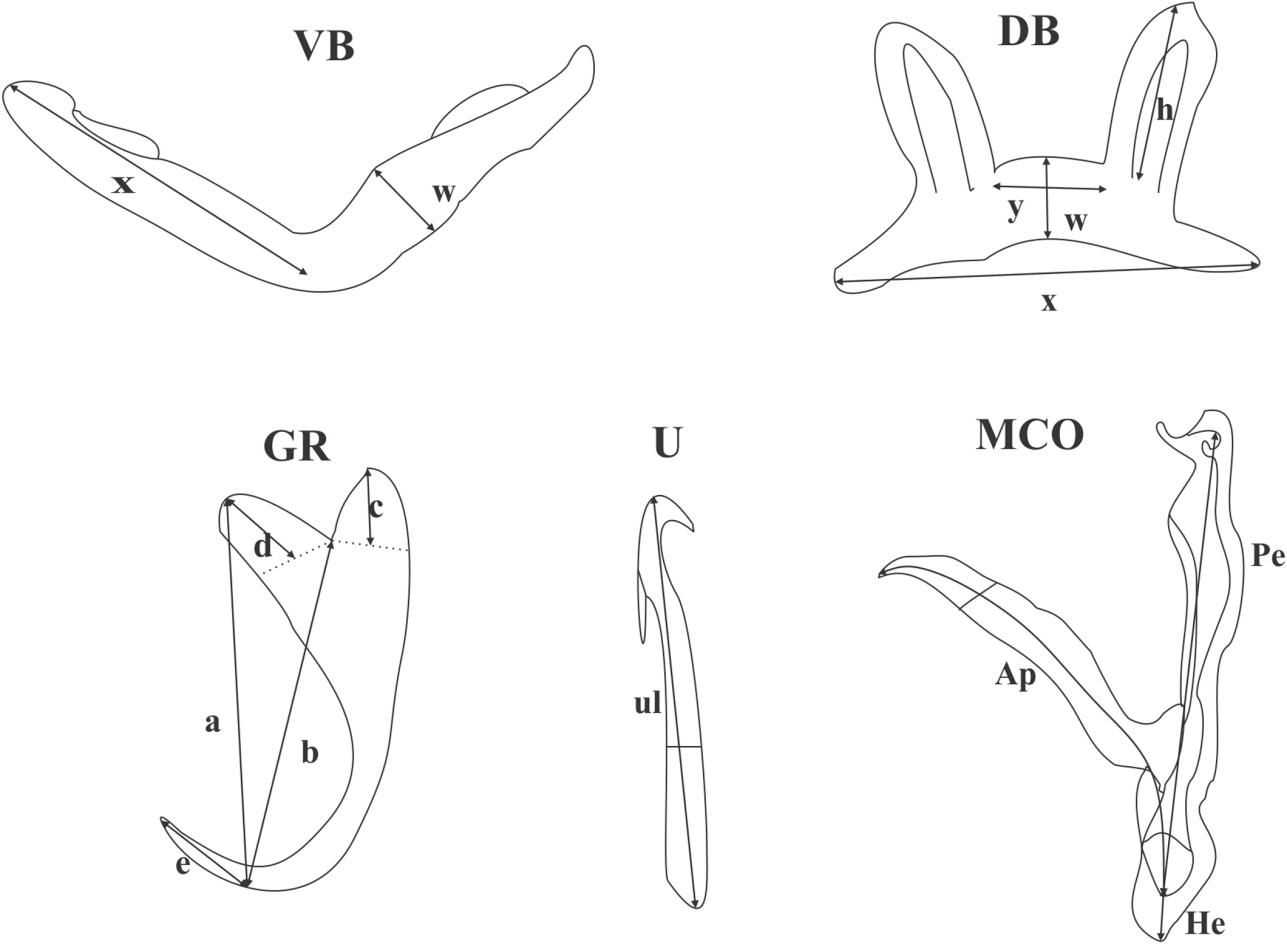
Measurements used in this study. Abbreviations: **VB** ventral transverse bar: **x** length of one ventral bar branch, **w** ventral bar maximum width; **DB** dorsal transverse bar: **h** length of auricle, **x** total length, **y** distance between auricles, **w** maximum width; **GR** gripus: **a** total length of gripus, **b** blade length, **c** outer root length, **d** inner root length, **e** point length; **MCO** male copulatory organ: **Ap** accessory piece length, **Pe** penis total length, **He** heel length; **U** uncinulus: **ul** uncinulus length.

Photomicrographs and measurements were obtained using a Zeiss Axioplan 2 Imaging light microscope with Axiovision 4.7.2 software; measurements were taken as proposed by Gussev [9]. The numbering of haptoral pieces (I-VII) was adapted after Euzet and Prost [5] and the method of naming is that proposed by Pariselle and Euzet [26]. Measurements were taken as shown in Figure 1 and are given in μm as Minimum − Maximum − Average (Standard Deviation) in Table 1. Data from this study were compared with the original species description by Douëllou [3]. Voucher specimens were deposited in the Iziko museum (SAMC-A089303; SAMC-A089304) and the Muséum National d'Histoire Naturelle, Paris, France (accession number MNHN HEL734 - HEL735).

**Table 1:** Measurement (in µm) of *Cichlidogyrus philander* parasitic on *Pseudocrenilabrus philander* in Padda Dam, compared to measurements provided by Douëllou (1993).

	Measurements (μm) Douëllou (1993) (n = 15)			Measurements (μm) Present study (n = 30)		
		
Structure	Min	Max	Ave.	Min	Max	Ave. (± SD)
Body:						
Length	260	400	321	252	473	341 (65)
Width	55	80	69	52	111	93 (11)
Pharynx *	-	-	-	17	20	19 (1)
Ventral bar (VB):						
w: maximum width of one bar branch	2	5	3	4	8	5 (1)
x: Length of one bar branch	25	29	28	19	39	29 (5)
Dorsal Bar (DB):						
h: Length of auricle	10	12	21	8	17	10 (2)
w: Maximum width	4	6	5	3	9	6 (1)
x: Total length	24	31	28	26	39	32 (1)
y: Distance between auricles	7	12	9	3	17	10 (3)
Ventral gripus (VB):						
a: Total length of gripus	28	32	30	29	33	31 (1)
b: Blade length	23	27	25	23	30	27 (2)
c: Outer root length	4	7	5	4	8	6 (1)
d: Inner root length	8	12	10	8	13	11 (1)
e: Point Length	8	11	10	5	12	9 (2)
Dorsal gripus (DG):						
a: Total length of gripus	29	36	33	29	39	34 (3)
b: Blade length	18	23	21	17	26	22 (2)
c: Outer root length	3	7	5	4	10	7 (2)
d: Inner root length	12	16	14	10	18	15 (2)
e: Point Length	6	9	7	4	9	7 (1)
Uncinuli (U) Length:						
I	22	24	23	15	27	23 (3)
II **	10	11	10	6	11	9 (1)
III ***(4)	19	20	16	11	22	17 (4)
IV ***(3)	15	17	20	13	24	19 (3)
V	22	25	23	21	28	24 (2)
VI	20	22	21	17	27	22 (2)
VII	18	20	19	17	25	20 (2)
MCO:						
Ap: Accessory piece length	27	35	32	27	47	40 (6)
He: Heel length	-	-	3	2	6	4 (1)
Pe: Penis Length	44	50	46	38	49	45 (3)

For SEM of the exterior of the parasite, ten specimens previously preserved in 70% ethanol were dehydrated in a graded series of ethanol and hexamethyldisilazane after Dos Santos *et al*. [1]. The dehydrated specimens were mounted on a strip of carbon conductive tape that was fixed to an SEM stub. Specimens were sputter coated with gold using an Emscope SC500 sputter coater (Quorum Technologies, Lewes, U.K.) and examined with a TESCAN Vega 3 LMH SEM (Brno, Czech Republic) at 6-10 kV acceleration voltage. For SEM of isolated hard parts, 20 parasites freshly removed from the gills of 5 fish were each placed individually on a concavity slide and digested with 0.5 μl of digestion buffer (9 parts ALT buffer: 1 part proteinase K) from a DNeasy^®^ Blood and Tissue kit (QIAGEN, Manchester, U.K.) after Dos Santos and Avenant-Oldewage [2]. Digestion of parasites was observed with a stereo microscope and to prevent the crystallisation of the digestion buffer, small volumes of distilled water were added. This addition of distilled water also allowed for residual buffer to be removed using a micropipette. Usually, 5-6 rounds of adding water and pipetting out the residual buffer and digested material were sufficient to wash out the residual buffer. The digested material and buffer were collected in a 1.5 µl microcentrifuge tube and stored for DNA analysis. The samples were then dried overnight in a Sanpla dry keeper desiccator cabinet (Kitaku, Osaka, Japan), sputter coated with gold, and examined using a Vega 3 LMH SEM at 6-10 kV.

### Molecular analysis

Using the digested tissue from specimens as outlined in the above procedure, genetic material was extracted using a DNeasy^®^ tissue kit (QIAGEN, Manchester, U.K.), according to the manufacturer's instructions. For this study, the large subunit of ribosomal DNA (28S), first internal transcribed spacer (ITS1) rDNA, and the mitochondrial cytochrome oxidase c subunit I (COI) fragments were used. Extracted genetic material from each specimen was used in triplicate for the 28S, ITS1 and COI amplifications, respectively. The 28S rDNA was amplified using primers C1 (forward; 5ʹ-ACCCGCTGAATTTAAGCAT-3ʹ) and D2 (reverse; 5ʹ-TGGTCCGTGTTTCAAGAC-3ʹ), according to the amplification protocol of Messu Mandeng *et* *al.* [21]. For ITS1, the ITS1A (5ʹ-GTAACAAGGTTTCCGTAGGTG-3ʹ) and ITSR3A (5ʹ-GAGCCGAGTGATCCACC-3ʹ) primers were used, and the primers ASmit1 (5ʹ-TTTTTTGGGCATCCTGAGGTTTAT–3ʹ) and Schisto3 (5ʹ- TAATGCATMGGAAAAAAACA–3ʹ) were used to target the COI region. The amplification protocol of Vanhove *et al.* [36] was used for both ITS1 and COI (excluding the use of nested PCR). Verification of successful amplicons was done on a 1% agarose gel, impregnated with GelRed^®^ (Biotium) and visualized with a UV transilluminator. For each marker, 10 amplicons were sequenced using standard BigDye chemistry, and analyzed on an ABI 3137 Automated Sequencer (Applied Biosystems, Foster City, CA, U.S.A). The obtained sequences were aligned and edited in MEGA 6 [35]. For the ITS1 and 28S sequences, 8 sequences from individuals whose trace files were of sufficiently good quality were phased using the default settings for the PHASED algorithm [33] in DnaSP v.5 [17] to produce 16 alleles. Closely related sequences (100) for *Cichlidogyrus* spp. and *Scutogyrus* spp. were obtained from GenBank using BLAST and aligned to the sequences produced in this study with MUSCLE [4] as implemented in MEGA 6 [35], followed by manual inspection. Sequences that covered less than 70% of the alignment were omitted from these analyses to improve accuracy. Pairwise distances between sequences were computed on MEGA 6 [35] using uncorrected *p*-distances. Identical sequences and haplotypes differing less than 0.01 [36] were removed. Phylogenies were reconstructed using the maximum likelihood (ML) method based on the Tamura-Nei model [34] with initial trees obtained by applying Neighbor-Join and BioNJ algorithms to a matrix estimated using the Maximum Composite Likelihood (MCL) approach; and the maximum parsimony (MP) method using the subtree-pruning-regrafting (SPR) algorithm [23]. Furthermore, 1000 bootstrap replicates were used to assess the robustness of all the resulting topologies. The analysis involved 33, 42, and 43 sequences with a total of 563, 239 and 320 positions in the final data sets for the 28S, ITS1 and COI markers, respectively. Sequences of the most distant *Cichlidogyrus* taxa from that of *C. philander* were used to root the phylogenies. The genetic distances and phylogenetic trees are presented in the appendixes to the manuscript (unidentified taxa removed from the distance tables for ease of representation).

## Results

### Light microscopy

**Figure 2:**
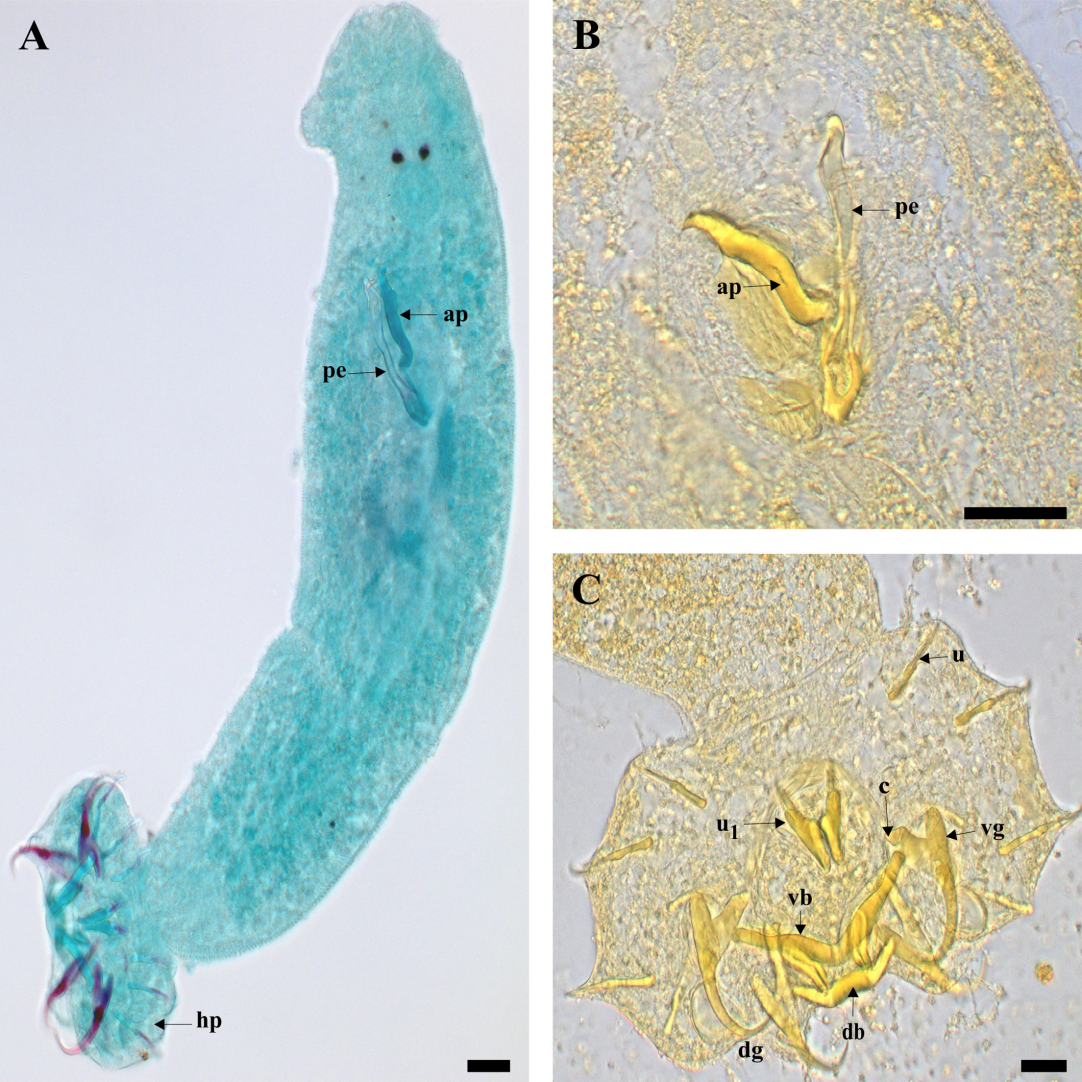
Light micrographs: **A** Whole mount of *Cichlidogyrus philander* stained with Horen's trichrome; **B** GAP-stained MCO; **C** GAP-stained haptor. Abbreviations: **ap** accessory piece; **c** cap; **db** dorsal bar; **dg** dorsal gripus; **hp** haptors; **pe** penis; **u** uncinulus; **u_1_**1st uncinulus; **vb** ventral bar; **vg** ventral gripus. Scale-bars: A-B, 20 μm; C, 10 μm.

The authors believe the drawings provided by Douëllou [3] are still sufficient and relevant, and were used as a reference. Measurements recorded are shown in Table 1 and are compared with those of Douëllou [3]. Using light microscopy, the male copulatory organ is seen to consist of a penis and accessory piece (Fig. 2A, B). The penis is straight and broad arising from a reduced basal portion with a heel. It is constricted at about 1/3 of its length, while the accessory piece is approximately as long and wide as the penis (Fig. 2A, B). The sclerotized parts of the haptor (Fig. 2C) are small with two transverse bars, two pairs of large gripi (anchors) and seven pairs of uncinuli (marginal hooks). The transverse bars are the ventral bar which is short, curved with slight constrictions towards extremities and a small dorsal bar that is slightly arched with two auricles. The gripi (anchors) are composed of the ventral and dorsal gripi, which have caps on the end of their roots. The ventral gripi are short with a narrow outer root and a wider inner root, a narrow base, long thin shaft, and long sharp point, while the dorsal gripi are longer and different in shape, with a slender base, short shaft and point and the outer root well developed. The seven pairs of uncinuli (hooklets) follow the basic morphology of monogenean marginal hooks, with each hook made up of three regions: a solid base, a relatively narrow, solid shaft, and a sickle-shaped termination [29]. These features agree with the initial description of the species using light microscopy.

### Redescription based on SEM

#### Male copulatory organ

**Figure 3:**
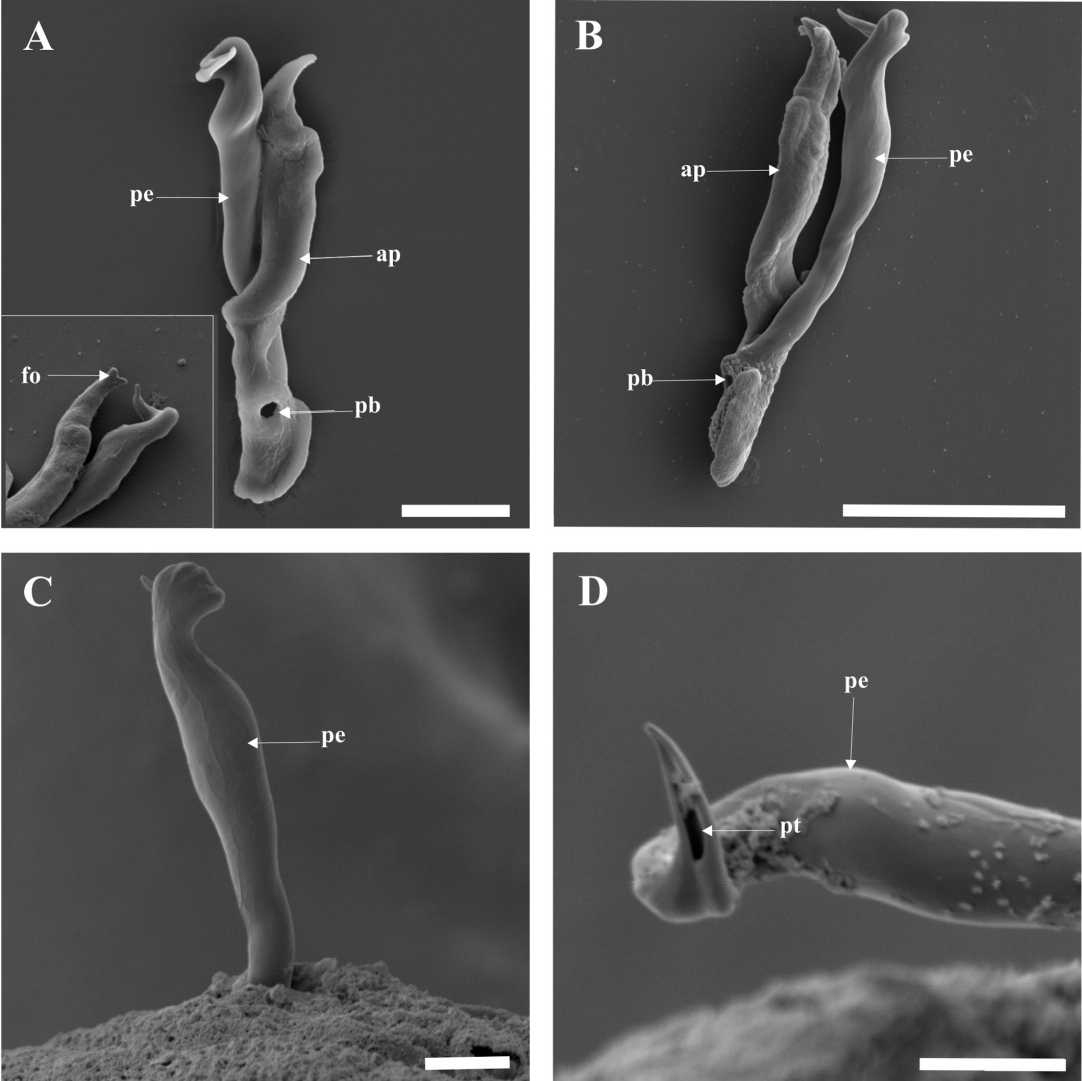
SEM micrographs: **A**, **A insert** and **B** MCO from digested tissue; **C** MCO within the tissue in lateral view; **D** peripheral region of MCO in contralateral view, showing opening at tip of penis. Abbreviations: **ap** accessory piece; **fo** forked terminal end of accessory piece; **pe** penis; **pt** opening at tip of penis; **pb** opening at base of penis. Scale-bars: A, A insert, 10 μm; B, 20 μm; C-D, 5 μm.

When digested, the male copulatory organ (MCO) shows the distal part of the penis forming a curve of almost 360°, which ends in a sharp, lateral termination. The MCO shows a distinct opening near its base (Fig. 3A, B). The accessory piece, however, has a hook-like extremity which may appear forked terminally (Fig. 3A insert). SEM of the exterior of the parasite (MCO within tissue), from the lateral view shows only the penis extending out of the parasites tissue with the penis looking like a tight fist (Fig 3C), while the contralateral view shows an opening at the midpoint of the lateral termination (Fig. 3D),

#### Haptoral sclerites

**Figure 4:**
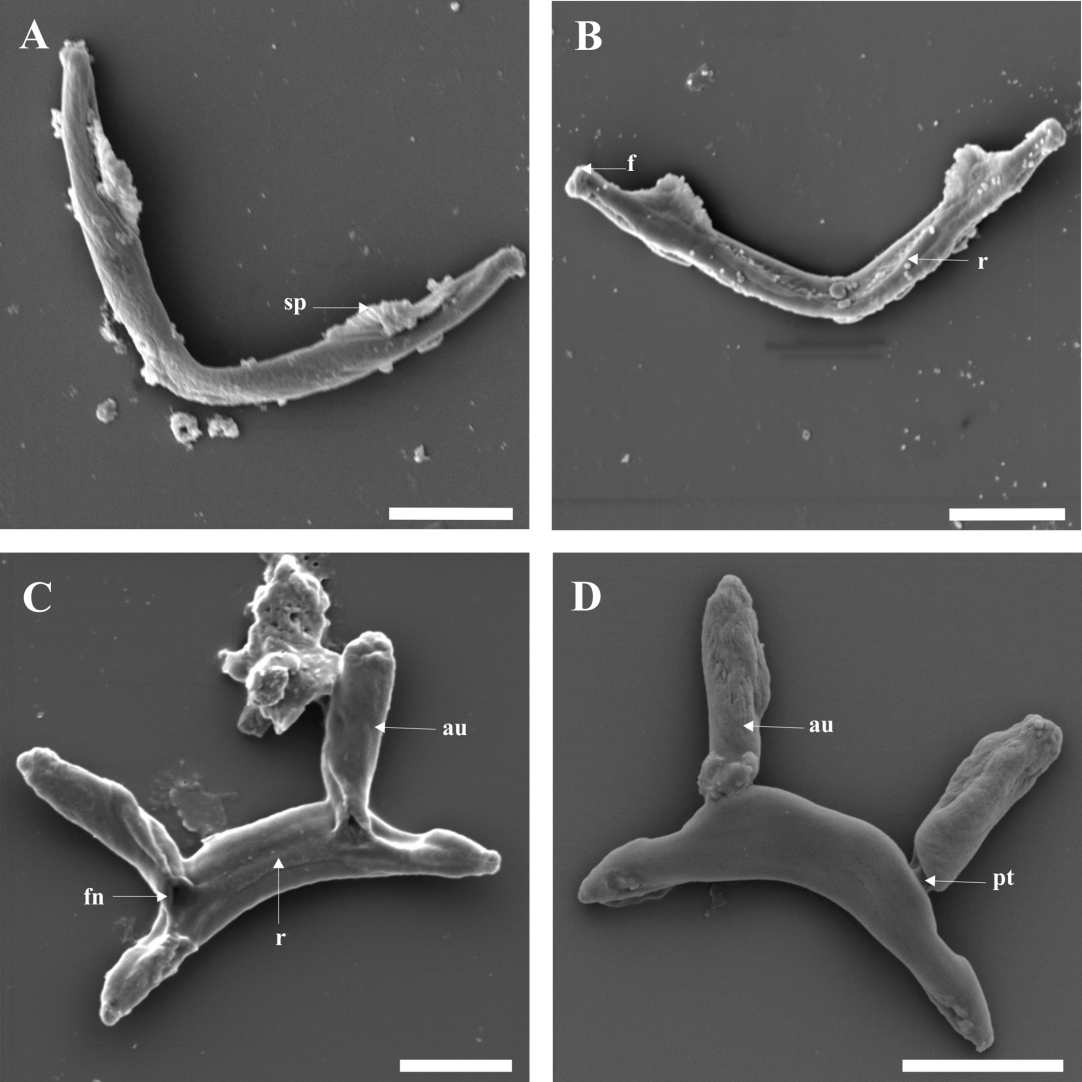
SEM micrographs of ventral and dorsal bars: **A** convex surface of ventral bar; **B** concave surface of ventral bar; **C** concave surface of dorsal bar; **D** convex surface of dorsal bar. Abbreviations: au auricle; f fold on extremities; **fn** fenestration; **pt** point of attachment of auricle; **r** rib; **sp** serrated plate. Scale-bars: 10 μm.

**Figure 5:**
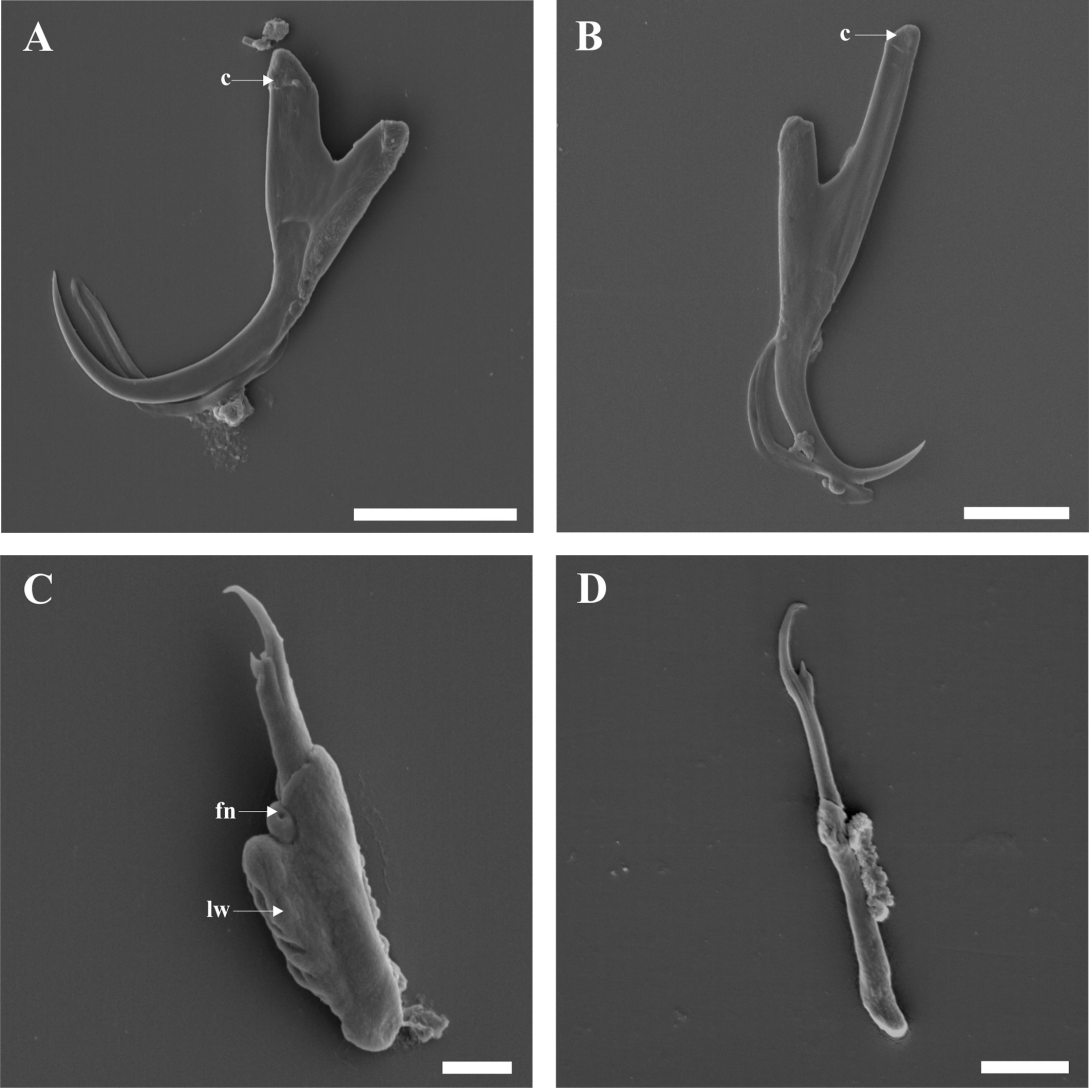
SEM micrographs of gripi (anchors) and uncinuli (hooklets): **A** ventral gripus: **B dorsal** gripus; **C** marginal hooklet I; **D** marginal hook. Abbreviations: **c** cap; **fn** fenestration; **lw** lateral wing. Scale-bars: A, B 10 μm; C, D 5 μm.

We were able to view both dorsal and ventral surfaces of the transverse bars. The dorsal (convex) surface of the ventral bar has serrated or tooth-like plates arising at the point of constriction near the extremities (Fig. 4A); these plates appear to be more serrated on the dorsal surface than on the ventral (concave) surface (Fig. 4B). The ventral surface shows a fold at the extremities, a depression and ribs (Fig. 4B). The ventral (concave) surface of the dorsal bar also has ribs and a fenestration at the base of each auricle (Fig. 4C); the dorsal (convex) surface lacks these structures but shows the point of attachment of the auricles to the dorsal bar (Fig. 4D). The first pair of uncinuli are stout and large and have a lateral wing on one side of their base; on top of the lateral wing, a ball-like structure with a small fenestration is visible (Fig. 5C). All sclerites were observed with SEM; however, no additional information was obtained for some of the structures as can be seen in Figures 5A, B and D.

#### Molecular analysis

For each marker, at least 8 of the 10 aplicons sequenced provided sequences of sufficiently good quality to allow for proper analysis (GenBank accession numbers: 28S MG279691-MG279698; ITSI MG250200-MG250207; COI MG288503-MG288510). Little to no variation was observed for these three markers, only polymorphic sites were present in the rDNA sequences and thus no intraspecific variation was seen for *C. philander*. The two analytical methods, ML and MP produced similar phylogenetic trees for the three markers. Therefore, the ML trees were used as representatives for both the 28S (Appendix 1.1) and ITS1 (Appendix 1.2), while the MP tree was used for COI (Appendix 1.3). Alignment with published sequences for the 28S rDNA showed an uncorrected *p*-distance of 3.16% − 6.15% between *C. philander* and other *Cichlidogyrus* taxa, with *Cichlidogyrus njinei* Pariselle, Bilong Bilong and Euzet, 2003 (HE792775) having the lowest, and *Cichlidogyrus pouyaudi* Pariselle and Euzet, 1994 (HQ010039) having the highest divergence value from *C. philander.* There was no intraspecific variation recorded for this marker. Interspecific distance values ranged from 0.18% − 8.08% (Appendix 2.1). The ITS1 region showed an uncorrected *p*-distance ranging from 12.97% to 20.08% between *C. philander* and other taxa, with an unidentified *Cichlidogyrus* sp. (KT037335) having the lowest sequence divergence, while another unidentified *Cichlidogyrus* sp. (KT037321) showed the highest sequence divergence from the study taxon. Intraspecific distances of 1.26% for *Cichlidogyrus tiberianus* Paperna, 1960 and 2.09% for *Cichlidogyrus acerbus* Dossou, 1982, and interspecific distances of 1.26% − 20.92% were observed for this maker (Appendix 2.2). For COI, the lowest uncorrected *p*-distance (20.31%) from *C. philander* was seen for *Cichlidogyrus sp.* (KT037383) and *Cichlidogyrus casuarinus* Pariselle, Muterezi Bukinga and Vanhove, 2015 (KX007849), while the highest sequence divergence (25.00%) was observed between the study taxon and an unidentified *Cichlidogyrus* sp. (KT037369). Intraspecific distances 3.13% (between *C. casuarinus* species) and interspecific distances of 1.25% to 25.31% were seen for this marker (Appendix 2.3). For 28S rDNA, *C*. *philander* forms a sister taxon to a clade containing most other taxa, while for the other two markers, this species groups sister to *C. casuarinus* (COI) and *Cichlidogyrus* sp. KT037335 (ITS1).

#### Diagnosis

Based on SEM study, *C. philander* was found to have a large penis that ends in an almost 360° curve, with an opening running from the midpoint of the sharp lateral termination to its base (Fig. 3A-D), and to lack a visible vagina. The ventral and dorsal bars have concave and convex surfaces (Fig. 4A-D) with ribs on the ventral surfaces (Fig. 4B, C), and the dorsal bar has fenestrations at the base of the auricles (Fig. 4C). The first pair of uncinuli displays a lateral wing on the left side of the base and a small fenestration on a ball-like structure on the lateral wing (Fig. 5C). These mentioned features were not visible with light microscopy. The sequence data for this taxon are also distinct from all other *Cichlidogyrus* species sequence data, based on the 28S rDNA, ITS1 rDNA and COI mtDNA.

## Discussion

Sclerotized structures such as the copulatory organs and haptoral parts remain the key features for most monogenean morphological taxonomy [12,20,28,30,38]. Therefore, it is of paramount importance to properly describe these structures, including their ultrastructural details. Exposing the hard parts by digesting away the surrounding tissue allows flattening of the sclerites and SEM examination of the surface details. The digested tissue also provides genetic material for molecular characterization [2,21].

In this study, *C. philander*, a minute parasite of *P. philander* [3,15], was redescribed. The redescription was based on SEM examination of exposed sclerites, revealing previously undescribed structures on the MCO, the transverse bars and the uncinuli I. For example, the distal part of the penis was shown to form a 360° curve and ends in a lateral termination with an opening near the middle (Fig. 3D). The opening at the base of the penis (Fig. 3A, B) is probably the entry point of ducts from the vesicula seminalis and prostatic reservoirs as suggested by Fannes *et al.* [7] for *C. casuarinus*, but their study did not show an opening at the tip of the penis. These two openings (at the tip and base of the penis) could be a single opening running from the tip to the base of the penis, which makes the penis a hollow organ as depicted in light microscopy (Fig 2A, B). Such details can be useful to differentiate species with similar MCOs. This was clearly shown in a study by Fannes *et al.* [8], which highlighted differences between the genitalia of *C. tiberianus* and *Cichlidogyrus dossoui* Douëllou, 1993. Another example is the differences observed on the concave surface of the dorsal bars of *C. casuarinus* in Fannes *et al.* [7], and *C. tiberianus* and *C. dossoui* in Fannes *et al.* [8] with *C. philander* in the present study. The concave surface of *C*. *casuarinu*s shows a round structure at the top of the fenestration found at the base of the auricle; *C. tiberianus* shows the same structure, but the structure is at the base of the auricle, while *C. dossoui* and *C. philander* lack this structure. Based on the haptoral configuration proposed by Vignon *et al.* [37], members of the genus *Cichlidogyrus* are placed in three main groups (groups A-C) that consists of a given combination of particular uncinuli. *C. philander* belongs to group B comprising *Cichlidogyrus* species that have a massive first pair of hooklets and smaller hooks in pair II − VII. The shape of the MCO and isolated haptoral parts corresponds well with the original drawings [3], showing that SEM study does not produce stronger deformations than the conventional light-optical methods.

Up to 90% of the digested tissue samples provided sufficient genetic material for amplification and successful sequencing. The uncorrected *p*-distances ranging from 3.16%–21.56% for the three markers confirm the distinctness of *C. philander* from other taxa of the genus. This is supported by the intra- and inter-specific distance ranges for each of the three markers, with the distance of *C. philander* from other taxa always within the interspecies boundaries. According to the ITS1 topology, the closest *Cichlidogyrus* taxon to *C. philander* is an unidentified *Cichlidogyrus* sp. (KT037335). Vanhove *et al*. [36] noted that this unidentified *Cichlidogyrus* sp. sequence and the *Cichlidogyrus zambezensis* Douëllou, 1993 sequence (COI) were both obtained from parasites collected from *Serraochromis robustus jalla* Boulenger, 1896 in Zambia. Thus, it seems likely that this unidentified sequence represents *C. zambezensis.* This would mean that *C. philander* and *C. zambezensis* may be closely related based on the ITS1 analysis. However, Vignon *et al*. [37] placed *C. philander* and *C. zambezensis* in different morphological groups. Importantly, in the COI topology, *C. philander* and *C. zambezensis* are not closely situated and no 28S sequence for *C. zambezensis* is currently available. Similarly, *C. casuarinus* is more closely situated to *C. philander* in the 28S and COI topologies than the ITS1. The large number of sequences not identified to species level makes it difficult to infer the phylogenetic relationships of *C. philander* meaningfully. Future studies incorporating additional sequence data, of particularly correctly identified taxa, and different analytical approaches could lead to more enlightening results.

### Conflict of interest

The authors declare that they have no conflict of interest.

## Figures and Tables

**Appendix 1.1:**
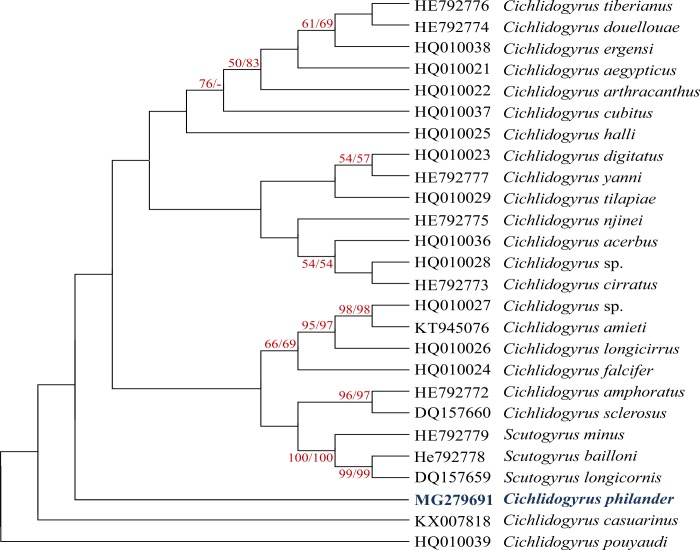
Bootstrap consensus tree based on maximum likelihood, inferred from analysis of the 28S rDNA fragment of *Cichlidogyrus philander* in relation to other taxa. Values along branches indicate percentage bootstrap support for maximum likelihood and maximum parsimony methods (ML/MP). Nodes without bootstrap values had support lower than 50% and were omitted or represented with a dash.

**Appendix 1.2:**
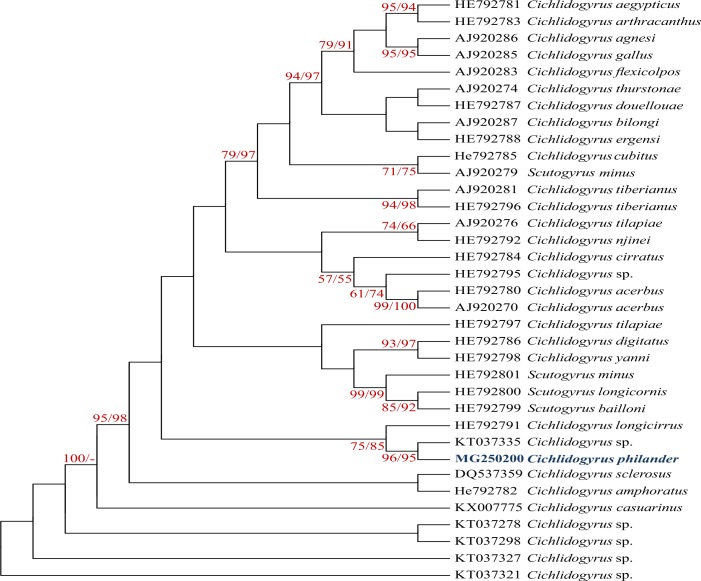
Bootstrap consensus tree based on maximum likelihood, inferred from analysis of the ITS1 rDNA fragment of *Cichlidogyrus* *philander* in relation to other taxa. Values along branches indicate percentage bootstrap support for maximum likelihood and maximum parsimony methods (ML/MP). Nodes without bootstrap values had support lower than 50% and were omitted or represented with a dash.

**Appendix 1.3:**
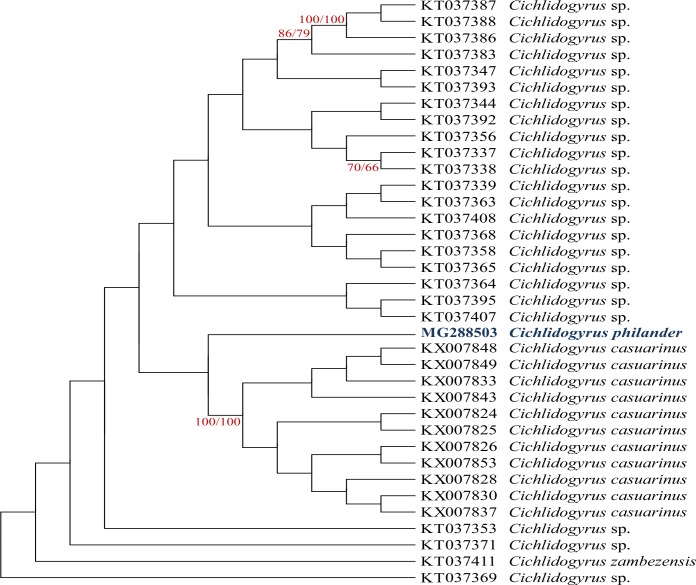
Bootstrap consensus tree based on maximum parsimony, inferred from analysis of the COI fragment of *Cichlidogyrus* *philander* in relation to other taxa. Values along branches indicate percentage bootstrap support for maximum likelihood and maximum parsimony methods (ML/MP). Nodes without bootstrap values had support lower than 50% and were omitted.

**Appendix 2.1:**
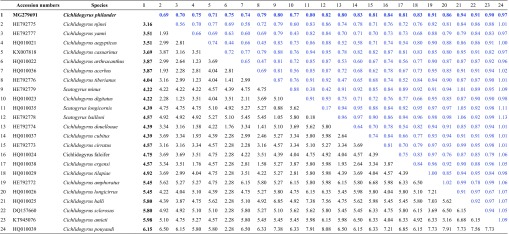
Genetic distances (%) between *Cichlidogyrus philander* and other *Cichlidogyrus* and *Scutogyrus* taxa based on the 28S rDNA marker (standard error estimates are shown above diagonal).

**Appendix 2.2:**
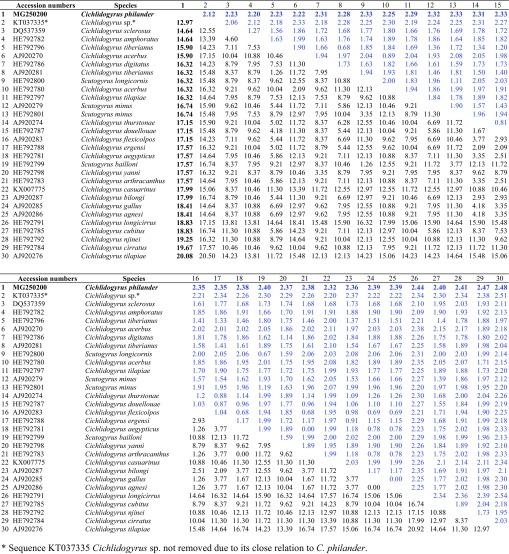
Genetic distances (%) between *Cichlidogyrus* *philander* and other *Cichlidogyrus* and *Scutogyrus* taxa based on the ITS1 rDNA marker (standard error estimates are shown above diagonal).

**Appendix 2.3:**
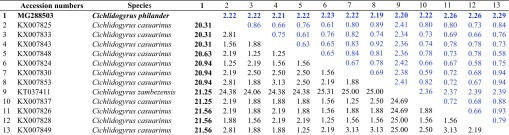
Genetic distances (%) between *Cichlidogyrus* *philander* and other *Cichlidogyrus* and *Scutogyrus* taxa based on the COI marker (standard error estimates are shown above diagonal).
